# Can a modified resuscitation course in a national psychiatric hospital improve medical provider confidence in medical emergency response

**DOI:** 10.1192/j.eurpsy.2025.2160

**Published:** 2025-08-26

**Authors:** Y. Y. Chew, N. Y. W. Lee, E. Seow, F. Yao

**Affiliations:** 1 Institute of Mental Health; 2 Khoo Teck Puat Hospital, Singapore, Singapore

## Abstract

**Introduction:**

Institute of Mental Health (IMH) is the only tertiary psychiatric hospital in Singapore and does not provide acute medical care. The on-call doctors, who are Advanced Cardiac Life Support (ACLS) certified, respond to medical emergencies. Resuscitation skills are expected to decay with time when not used frequently and thus can pose an important challenge to maintain the doctors’ skills in settings with low volumes of code blue situations (Au *et al.* Resuscitation 2009;138:284-296). Our code blue aduits revealed significant competency gaps. IMH introduced a biannual resuscitation training program which includes a video demonstration of the optimal code blue response, hands-on session to review airway management techniques, operation of the defibrillators used in IMH, recognition and management of cardiac arrest rhythms including a pre-course ECG worksheet, familiarisation with the emergency drugs used in IMH, and a code blue drill. Due to COVID-19, the original course was shortened by removing the video demonstration and code blue drill, augmenting the home-based question paper with IMH-specific clinical vignettes.

**Objectives:**

We aimed to determine the common conditions resulting in code blue activations and whether the modified course was equivalent to the original course or ACLS in maintaining resuscitation currency and doctors’ confidence in responding to emergency scenarios.

**Methods:**

Data was collected from June to August 2023 with consent via an electronic feedback form, to reduce non-response bias, from doctors who have responded to code blue activation in IMH. Qualitative justification on the responses were collated. Efforts were made to collect at least 25 responses from doctors with different levels of experience to minimize sampling bias. Surveys were anonymised, questions were vetted by 2 senior doctors and the survey was kept short to reduce response bias. Binary responses were tabulated for analysis and content analysis was done for feedback obtained.

**Results:**

Of 28 respondents, most were Psychiatry trainees (60.7%) with 1-2 years of experience working in IMH (36.7%) and more than 30 overnight duties (53.6%). The most commonly encountered emergency scenarios were hypotension (31%) and desaturation (20%). 92.9% of participants agreed that the modified course was useful for emergency scenarios faced. 53.6% of participants attended both the full and modified course, amongst whom, 60% reported that the modified course was equivalent to the full course. Only 50% felt that ACLS alone would suffice. Qualitative feedback obtained from participants reiterated that it was a context-specific and timely refresher course.

**Conclusions:**

IMH doctors were satisfied with the modified resuscitation course and found it effective for frequently encountered emergency scenarios suggesting it as a valuable training adjunct in low code blue volume settings.

**Disclosure of Interest:**

None Declared

